# RUNX1-ETO: Attacking the Epigenome for Genomic Instable Leukemia

**DOI:** 10.3390/ijms20020350

**Published:** 2019-01-16

**Authors:** Emiel van der Kouwe, Philipp Bernhard Staber

**Affiliations:** Department of Internal Medicine I, Division of Hematology and Hemostaseology, Medical University of Vienna, 1090 Vienna, Austria; emiel.vanderkouwe@meduniwien.ac.at

**Keywords:** RUNX1, AML1, ETO, RUNX1-ETO, AML1-ETO, leukemia, epigenetic, methylation, histone, enhancer, chromatin, conformation

## Abstract

Oncogenic fusion protein RUNX1-ETO is the product of the t(8;21) translocation, responsible for the most common cytogenetic subtype of acute myeloid leukemia. RUNX1, a critical transcription factor in hematopoietic development, is fused with almost the entire ETO sequence with the ability to recruit a wide range of repressors. Past efforts in providing a comprehensive picture of the genome-wide localization and the target genes of RUNX1-ETO have been inconclusive in understanding the underlying mechanism by which it deregulates native RUNX1. In this review; we dissect the current data on the epigenetic impact of RUNX1 and RUNX1-ETO. Both share similarities however, in recent years, research focused on epigenetic factors to explain their differences. RUNX1-ETO impairs DNA repair mechanisms which compromises genomic stability and favors a mutator phenotype. Among an increasing pool of mutated factors, regulators of DNA methylation are frequently found in t(8;21) AML. Together with the alteration of both, histone markers and distal enhancer regulation, RUNX1-ETO might specifically disrupt normal chromatin structure. Epigenetic studies on the fusion protein uncovered new mechanisms contributing to leukemogenesis and hopefully will translate into clinical applications.

## 1. Introduction

An impaired genomic maintenance machinery and increased mutation rates confer a selective advantage to cancer cells resulting in their outgrowth [1]. The t(8;21)(q22;q22) translocation, first described in 1973 [2], is one of the most common chromosomal aberrations in acute myeloid leukemia (AML) [3]. Albeit described in a broad range of leukemia [4,5], the abnormality is predominantly found in the M2 subtype of AML according to the French-American-British classification with blasts displaying signs of neutrophilic differentiation [6]. The translocation between chromosomes 8 and 21 fuses Runt-related transcription factor (RUNX) (RUNX1) with Eight-Twenty-One (ETO) and generates the RUNX1-ETO oncoprotein. RUNX1, belongs to the RUNX family of genes, mammalian homolog of Drosophila runt [7,8], and is an essential hematopoietic transcription factor which constitutes the DNA-binding subunit of the heterodimeric core-binding factor (CBF) complex. RUNX1 associates to the non-DNA-binding factor CBFβ; chromosomal translocations and inversions on either of the two transcription factors represent the so-called CBF-AMLs.

For the induction of leukemia, RUNX1-ETO expression requires additional secondary genetic alterations [9,10]. Here, we review and discuss the mechanisms how the fusion protein can perturb the epigenome to induce a leukemic state. 

## 2. RUNX1 Transcription Factor

### 2.1. RUNX1 and Hematopoiesis

RUNX1, also known as Acute Myeloid Leukemia 1 (AML1), Core-Binding Factor-alpha-2 (CBFa2), and Polyoma Enhancer-Binding Protein-2alphaB (PEBP2aB), is a key transcription factor in hematopoietic development, and was first identified in 1991 [11]. Studies on mouse embryology found that RUNX1 is expressed in progenitors of primitive hematopoietic cells [12,13] which emerge from the yolk sac shortly after gastrulation [14,15] and marks the maturation of pre-hematopoietic stem cells (pre-HSCs) [16,17]. RUNX1 is expressed in all sites of blood formation and is required in definitive hematopoiesis, the continuous production of mature blood cells during the entire adult life span [18,19]. Generation of RUNX1 homozygous and heterozygous knockout mouse models played a key role in determining its function in hematopoiesis. Removal of RUNX1 in homozygous knockout mice showed fetal death at E12.5 because of hemorrhaging along the central nervous system and the lack of definitive hematopoiesis [20,21,22]. Although RUNX1 heterozygous mice survive into adulthood with minor hematological defects [21,23], definitive hematopoiesis is perturbed in a spatial and temporal manner [24,25]. Importantly, RUNX1 alone is insufficient for hematopoiesis and cooperates with additional lineage-specifying transcription factors such as members of the ETS [26,27] and GATA [28] family. Thus, RUNX1 is crucial for the maturation of a wide range of hematopoietic cells including, but not limited to, myeloid blood cells [29,30], B-cells [31,32], and T-cells [33,34].

Altered function of RUNX1 by intragenic mutations or chromosomal rearrangements in myeloid neoplasms provides evidence of the pivotal role of this transcription factor in hematopoiesis. Over 50 translocations affecting RUNX1 are reported for leukemia which result in fusion proteins involved in a broad spectrum of malignant diseases [35]; Most recurrent examples include RUNX1-ETO, RUNX1-EVI1, and ETV6-RUNX1, respectively the products of t(8;21)(q22;q22), t(3;21)(q26.2;q22), and t(12;21)(p13;q22) translocations. Also, around 60 different mutations in RUNX1 were reported, mostly frameshift mutations as a product of insertions and deletions in the coding sequence [36]. A striking feature of RUNX1 alterations is the mutual exclusivity of RUNX1 sequence mutation and chromosomal aberrations in leukemia [37] which we will discuss later.

### 2.2. RUNX1 Structure

**Figure 1:**
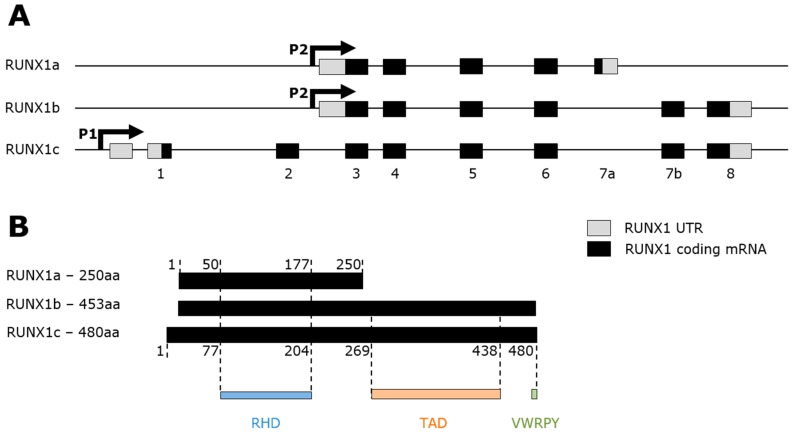
The three main RUNX1 isoforms, genomic locus and protein domains. (**A**) Schematic diagram of RUNX1a, RUNX1b and RUNX1c splicing variants on chromosome 21. RUNX1a and RUNX1b are transcribed from the P2 promoter and contain respectively 5 and 6 exons. RUNX1c is transcribed from the P1 promoter and contains 8 exons. (**B**) Schematic diagram of RUNX1a, RUNX1b and RUNX1c protein products from the alternative splicing and their protein domains. RUNX1a contains only the RHD whereas RUNX1b and RUNX1c contain the RHD, TAD and VWRPY motif.

At the transcriptional level, three major isoforms of RUNX1 are transcribed, RUNX1a, RUNX1b, and RUNX1c, and are the product of two promoters and alternative splicing (Figure 1A). RUNX1a and RUNX1b, respectively responsible of generating 250 and 453 amino-acid-long proteins, are transcribed from a proximal promoter P2. RUNX1c, on the other hand, comes from a distal promoter P1 and translates into a protein of 480 amino-acids (Figure 1B). Splicing variants of RUNX1 are strictly regulated in hematopoiesis as all three isoforms are expressed in a temporal and tissue-specific manner.

RUNX1a, enriched in the immature fraction of cord blood cells [38], promotes hematopoietic differentiation of human pluripotent stem cells [39]. RUNX1a and RUNX1b are shown to be present consistently throughout hematopoietic differentiation [40,41]. RUNX1c is involved in definitive hematopoiesis in a human embryonic stem cell in vitro model [40]. Interestingly, recent work in mice suggested an important balance between RUNX1b and RUNX1c for megakaryocyte and erythrocyte differentiation [42].

Noteworthy, RUNX1a lacks the transactivation domain (TAD) in the C-terminal region of the protein structure. The TAD contains the activating and inhibitory domains as well as the evolutionarily conserved VWRPY penta-peptide motif that interacts with mammalian homolog of Groucho, or transducin-like enhancer of split (TLE1) [43], a DNA-binding corepressor family of genes involved in hematopoietic differentiation. However, both TAD and VWRPY domains are present in RUNX1b and RUNX1c. More importantly, all three RUNX isoforms share the same 128 amino-acid Runt homology domain (RHD) at the N-terminal region which mediates binding to the TGt/cGGT consensus sequence [44] and the interaction with its heterodimerization partner core-binding factor beta (CBFβ). 

### 2.3. RUNX1 and the CBFβ Complex

The RUNX family of genes (RUNX1, RUNX2, and RUNX3) shares a non-DNA-binding partner core-binding factor beta (CBFβ), ubiquitously expressed and encoded by a single gene in mammals [45,46]. CBFβ increases the RUNX DNA-binding affinity and complex stabilization. Despite that RUNX1 can bind DNA as a monomer in vitro, heterodimerization with the non-DNA binding subunit CBFβ triggers flexible DNA-recognition loops, thus stabilizing the complex and increasing RUNX1 binding to DNA [47,48,49]. Cooperation between RUNX1 with CBFβ regulates ubiquitin-mediated degradation of RUNX1 [50] and enhances RUNX1 phosphorylation/acetylation responsible for a decreased interaction with transcriptional repressors [51]. Post-transcriptional regulation of both partners increases the ability of RUNX1 to activate transcription. The RUNX1–CBFβ complex interacts with PU.1, C/EBPα, p300, mSin3a, GATA1, and Fli1 [52,53,54,55,56] and is able to regulate essential classes of proteins such as growth factors (GM-CSF, MPO and IL-3), surface receptors (TCRA, TCRB, M-CSF receptor and FLT3), signaling molecules (CDKN1A), proliferation and survival regulators (BLK and BCL2), and transcription activators (STAT3 and MYB) [57,58]. Chromosomal translocations and inversions that target the transcription factors RUNX1 and CBFβ constitute the group of core-binding factor acute myeloid leukemias, CBF-AMLs.

## 3. RUNX1-ETO Fusion Protein

### 3.1. t(8;21) AML

Molecular cloning of the translocation breakpoints identified the RUNX1-ETO protein (also known as AML1-ETO, RUNX1-MTG8, and RUNX1-RUNX1T1), a product of the rearrangement of RUNX1 on chromosome 21 with ETO on chromosome 8 [11,59]. The t(8;21)(q22;q22) translocation is the third most common genetic alteration and the most common chromosomal aberration in de novo AML [3] and is found at a frequency of 7% in adults [3] and 12% in pediatric patients [60]. De novo t(8;21) AMLs have a relatively favorable prognosis with a high complete remission rate and long disease-free survival [61,62]. After treatment for hematological malignancies and solid tumors, secondary AML with t(8;21) translocations can occur which represents 10–20% of all t(8;21) AML cases [63,64].

### 3.2. RUNX1-ETO Structure and Repressive Activity

**Figure 2:**
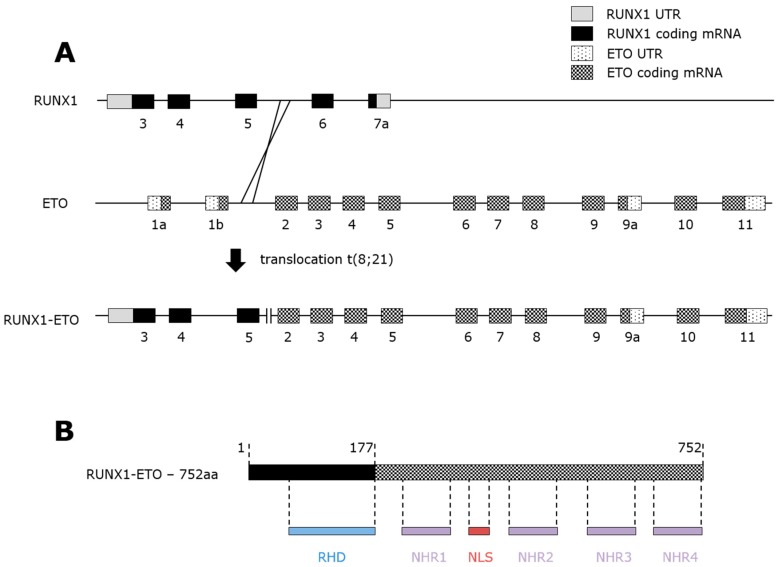
RUNX1-ETO structure and protein domains. (**A**) Schematic diagram of the t(8;21) translocation between the intron 5 of RUNX1 and the intron 1 of ETO. RUNX1-ETO contains 3 exons from the RUNX1 gene and either 9 or 11 exons from the ETO gene. (**B**) Schematic diagram of full-length RUNX1-ETO with the protein domains. The fusion protein has the Runt Homology Domain (RHD), four Nervy Homology Regions (NHRs) and the Nuclear Localization Signal (NLS).

ETO, also known as RUNX1 Translocation partner 1 (RUNX1T1) and Myeloid Transforming Gene on chromosome 8 (MTG8), contains a total of 14 exons including two alternative splicing variants, ETO9a and ETO11a [65,66], and four conserved domains termed nervy homology regions (NHRs) numbered 1 to 4 [67]. At the moment of its discovery, ETO was shown to lack DNA-binding ability [68] but to harbor transcriptional repression domains [69]. The four evolutionarily conserved nervy homology domains (NHR1-4) mediate ETO’s ability to interact with other proteins, mostly repressors [69,70,71]. All four NHRs interact with a wide range of regulatory proteins. The NHR1 region, sharing a homologous domain to TATA binding protein-associated factors (TAFs) [72], interacts with activation domain 1 (AE1) of E proteins like HEB and E2a. The interaction leads to an E protein-mediated transcriptional inhibition through displacement of P300/ CREB-binding protein (CBP) coactivators [73,74]. Most of RUNX1-ETO biochemical properties in leukemogenesis revolve around NHR2. NHR2-mediated oligomerization with corepressors like the nuclear co-repressor protein/silencing mediator of retinoic acid and thyroid hormone receptor (N-CoR/SMRT), mSin3a, and histone deacetylases (HDACs) [69,75,76]. NHR3 contains a coiled-coil structure which could help recruit transcriptional factors and NHR4 has two zinc-finger domains shown to recruit N-CoR/SMRT/HDAC transcriptional repressors [77]. Finally, between NHR1 and NHR2, ETO possesses a nuclear localization signal (NLS) [78,79]. The chromosomal breakpoints to generate RUNX1-ETO occur in intron 5 of the RUNX1 locus and in intron 1 of the ETO locus [80,81] (Figure 2A). Although the exact origin and mechanism that drive the t(8;21) translocation remain unclear, Wnt/βcatenin signaling has been suggested to promote genomic proximity between RUNX1 and ETO genes [82]. With a total of 752 amino-acids, RUNX1-ETO is constituted of the N-terminal end of RUNX1 (177 amino-acids) containing the RHD [59], and almost the complete ETO frame (575 amino-acids) (Figure 2B).

After t(8;21) translocation, RUNX1 is fused to ETO which generates the full-length RUNX1-ETO contains RUNX1 exons 1-5 and ETO exons 2-11, and all four NHRs were conserved which explained why early work on RUNX1-ETO focused on its repressive function. NHR2 has been proven essential for leukemogenesis [76,83], its disruption impairs the self-renewal ability of RUNX1-ETO in hematopoietic progenitors [84]. Because of the two alternative splicing variants of ETO, the t(8;21) translocation leads to two RUNX1-ETO splicing isoforms: RUNX1-ETO9a and RUNX1-ETO11a. Respectively lacking the NHR3-4 and the NHR4 domains at the protein level, RUNX1-ETO9a displays a lower capacity to inhibit native RUNX1 [85] but its leukemogenic potential is stronger compared to the unspliced form in mice [86].

### 3.3. RUNX1-ETO Cooperation with RUNX1

In recent years, increased evidence has emerged about the requirement of RUNX1 in leukemia cells with RUNX1-ETO. Next-generation sequencing in clinical studies revealed that mutations in RUNX1 did not occur in t(8;21) AML patients and that an active form of RUNX1 is maintained [37,87]. Consistent with this concept, RUNX1 knockdown in Kasumi-1 cells resulted in apoptosis and can be rescued with subsequent RUNX1-ETO knockdown [88]. Likewise, in a model for RUNX1-ETO leukemogenesis with human CD34+ cells, RUNX1 was required for cellular growth [89]. Recently, we demonstrated that HSCs require a functional RUNX1 to maintain adequate PU.1 levels, which is critical for RUNX1-ETO leukemia development in mouse transplantation models [90]. Using a broad range of truncated RUNX1 gene together with chromatin immunoprecipitation assays, RUNX1 was shown to be part of the RUNX1-ETO transcription factor complex [91].

### 3.4. RUNX1-ETO Decreases DNA Repair Capabilities and Compromises Genomic Stability

In somatic cells, DNA damage repair mechanisms are essential for genome integrity and cell viability [92]. Decreased levels of DNA repair capabilities could result in the accumulation of chromosomal aberrations and genomic instability. Acute myeloid leukemia, characterized by the clonal expansion of immature hematopoietic cells in the bone marrow, presents mutations in DNA damage response (DDR) genes which can affect disease progression and therapy resistance [93,94,95].

Increasing evidence suggests that early presence of RUNX1-ETO may promote mutagenesis. Several studies showed that RUNX1-ETO downregulates genes involved in base-excision repair mechanism (BER) [96,97,98]. OGG1, 8-oxoguanine DNA glycosylase, is required in the BER pathway to remove oxidized guanine nucleotides exposed to reactive oxygen species. Ectopic expression of the RUNX1-ETO in cell lines and hematopoietic stem and progenitor cells showed that OGG1 is downregulated [96,97] as well as in t(8;21) AML patients [99]. Similarly for genes involved in homologous recombination-associated genes, BRCA2, and ATM, a core DNA repair and cell cycle regulator, are also found downregulated in RUNX1-ETO-expressing cell lines [98]. Similarly, recent work in mouse with transduced RUNX1-ETO bone marrow reported suppressed ATM, BRCA1, BRCA2 and RAD51 as responsible for higher levels of DNA damage [100]. Interestingly, C-terminal end-deleted RUNX1 shows repressed expression of Gadd45a, a sensor of DNA stress [101]. This suggests that RUNX1, under physiological condition, plays a key role in monitoring cell stress to maintain genomic stability. One possible conclusion is that altered RUNX1 and RUNX1-ETO fusion protein compromise genome integrity by impairing DNA stress monitoring and DNA repair capabilities. 

In line with previous statements, RUNX1-ETO cells display high levels of DNA stress and are accompanied by elevated p53 and associated target genes which leads to the induction of apoptosis [97]. Unsurprisingly, knockdown of p53 using short hairpin RNA (shRNA) revealed increased resistance of AML1-ETO cells to radiation and chemotherapy [97] but was also associated with worse prognosis in mice [102]. AML with the t(8;21) translocation shows good response to currently available treatments, probably because of the synergistic effect with elevated p53 levels subsequent to DDR gene alteration and increased DNA stress. Although it seems paradoxical that the RUNX1-ETO oncogene is capable of apoptosis induction, this mechanism could protect leukemic clones from further accumulation of DNA damage, which could otherwise lead to cell exhaustion. Interestingly, AML cells able to excessively repair DNA damage are implicated in disease progression and therapeutic resistance [99,103]. Seemingly contradictory, lowered DNA repair capability enables RUNX1-ETO clones to handle high cellular DNA damage which consequently ensures self-renewal and provides flexibility to prevent cell exhaustion.

On the other hand, excessive DNA repair capabilities could contribute to disease progression and treatment resistance. In t(8;21) AML patient samples, higher levels of BRCA1, RAD51, and CHEK2 genes expression showed worse prognosis and reduced survival [103]. Similarly, increased OGG1 levels displayed a significant reduction in overall survival and an increased risk of relapse [99]. A possible explanation could be that increased DNA repair capabilities of RUNX1-ETO cells fails to trigger p53-mediated apoptosis which in turn leads to a weaker response to available treatments. 

Impaired defense mechanism against DNA damage compromises genomic integrity and leads to secondary chromosomal abnormalities. Indeed, chromosomal aberrations are detected in 70% of t(8;21)-positive AML [104,105]. Loss of sex chromosome (-X or -Y) is predominantly found (47%), followed by the long arm deletion of chromosome 9, del(9q) (15%) and trisomy 8 (6%) [105]. Of note, the common region to X and Y chromosomes PAR1 contains the GM-CSF receptor α subunit (CSF2RA) gene which is found as a tumor suppressor gene in mouse transplantation model [106]. Similarly, TLE1 and TLE4 were suggested as potential tumor suppressors located in the lost genomic region in del(9q) [107] which in turn might be a poor prognostic factor in t(8;21) AML [108,109]. Current state of knowledge suggests that reduced DNA repair regulators in the presence of RUNX1-ETO play a role in the genomic instability and pathogenesis of t(8;21) AML which could facilitate the acquisition of cooperating secondary events. 

Although several chromosomal aberrations associated with RUNX1-ETO fusion protein have been described, it is interesting to note that normal karyotype-AML (NK-AML) is correlated with high native RUNX1 levels. NK-AMLs, representing 40% of all AML [3], are associated with poorer clinical outcome and event-free survival compared to CBF leukemia [110]. Interestingly, these NK-AML patients have similar outcome as t(8;21) AML with high levels of DDR genes [99,103]. It is therefore possible that unaltered RUNX1 maintains DNA damage repair capabilities of the cell and leads to therapeutic resistance. Therefore, and given the good prognosis of t(8;21) AML, it is tempting to hypothesize that the presence of RUNX1-ETO sensitizes the cell to available therapeutics. Moreover, decreased DNA repair capabilities could generate a mutator phenotype, which in turn explains the extensive available literature on RUNX1-ETO-associated mutations, especially epigenetic regulators involved in DNA methylation [4,5].

## 4. RUNX1-ETO is Associated with Altered Methylation

DNA methylation represents a key mechanism of gene regulation in mammalian cells [111]. During hematopoiesis, stem cells give rise to multi-potent progenitors which subsequently undergo commitment to mature blood cells. The patterns of DNA methylation at cytosine residues within CG dinucleotides (CpG) islands govern at the epigenetic level the dynamic changes in gene expression required for the maturation of hematopoietic cell lineages [112,113,114]. The net effect of this modification is to induce a closed chromatin configuration, resulting in stable gene silencing.

Recent work showed that ectopic expression of RUNX1 activates genes by DNA demethylation [115]. RUNX1 shares a genome-wide co-occupancy with ten-eleven translocation-2 (TET2), which converts 5-methylcytosine (5mC) to 5-hydroxymethylcytosine (5hmC) to promote DNA demethylation and gene activation [116]. The cooperation of both factors plays a key role in normal hematopoietic development by enhancer demethylation. Interestingly, TET2 is mutated with high frequency in patients suffering from a wide variety of hematopoietic diseases [117] including AML [118,119] and t(8;21) AML [120]. Similarly, loss of TET2 in the presence of RUNX1-ETO showed a genome-wide increase in DNA methylation at active enhancer regions and promotes leukemia in mouse transplantation models [121]. It is evident that RUNX1-ETO together with mutated TET2 disrupts the methylation at regulatory regions and thus the expression of important genes in hematopoietic development. A similar conclusion can be drawn for isocitrate dehydrogenase (IDH) proteins which, under normal conditions, supply NADPH necessary for lipid biogenesis and protection from oxidative and radiation-induced damage [122]. However, mutant IDH enzymes produce high levels of the oncometabolite (R)-2-hydroxyglutarate that competitively inhibits dioxygenase enzymes which in turn modifies 5mC to 5hmC. This promotes DNA demethylation and gene activation [116] in the same manner as TET2 would. Interestingly, IDH1 and IDH2 mutations are almost mutually exclusive with loss-of-function mutations in TET2 [123,124]. IDH1 and IDH2 mutations have been found in 5% of RUNX1-ETO AML [105]. Additionally, DNA methyltransferases (DNMT1, DNMT3a, and DNMT3b), which catalyze the CpGs methylation [125,126], are also mutated along the fusion protein. RUNX1-ETO is able to recruit DNMTs to the regulator regions of its target genes and has been shown to interact with DNMT1 for transcriptional repression [127] but also to recruit DNMT3a in cooperation with HIF1α [128,129]. Consistent with RUNX1-ETO cells adopting a mutator phenotype, RUNX1-ETO is shown to interact with a broad range of methylation regulators, namely DNMT, TET, and IDH, which are often found mutated in t(8;21) AML [105,120].

## 5. RUNX1-ETO Recruits Histone Markers

On a genome-wide level, the mechanism by which RUNX1-ETO interferes with native RUNX1 in normal hematopoiesis remains poorly understood. Although several early studies on RUNX1-ETO described its repressor function, the fusion protein is also capable of gene activation. Albeit conceptualized as the scaffold that packages DNA, histones actively participates in gene regulation and epigenetic function [130]. RUNX1-ETO, for both activation and repression of genes, heavily relies on histone modulation which modifies chromatin structure.

Early work showed the dominant-negative regulatory effect of RUNX1-ETO on the transcription of essential RUNX1 targets for normal myeloid differentiation [131]. As described above, RUNX1-ETO contains the four conserved NHR motifs from the ETO moiety which binds repressive complexes such as N-CoR/SMRT [71,132]. More importantly, the fusion protein alters the chromatin structure through its interaction with HDACs [71,132]. By removal of acetyl group from histones, HDACs create a closed chromatin conformation state preventing gene transcription. Yeast two-hybrid and chromatin immunoprecipitation assays revealed the importance of NHR2 and NHR4 domains in the interactions of ETO and RUNX1-ETO with HDAC1, HDAC2, and HDAC3 [70,77,133,134]. Interestingly, studies using HDAC inhibitors, Trichostatin A and valproic acid, were shown in cell lines to block the capabilities of RUNX1-ETO to suppress myeloid differentiation [133,135]. These studies demonstrate that the repressive function of RUNX1-ETO by HDAC recruitment is an important mechanism for its leukemogenic properties.

On the other hand, both RUNX1 and RUNX1-ETO are also capable of recruiting the histone acetyltransferase (HAT) p300/CBP complex. The latter changes the local nucleosomal environment by acetylating histone lysine residues and recruiting the transcriptional machinery thus playing an essential role in gene regulation in normal hematopoiesis [136,137,138]. In cell lines with ectopic p300 overexpression, RUNX1 is shown to bind p300 which stimulates RUNX1-dependant transcription and induces myeloid differentiation. The same study demonstrates that the RUNX1 protein domain starting outside the RHD up to the C-terminal end mediates the interaction with p300 [55]. Recruitment of p300 acetyltransferase by RUNX1 fulfills two purposes: self-acetylation for enhanced activity [139] and histone acetylation [140]. Interestingly, although RUNX1-ETO completely lacks the C-terminal end responsible for p300 interaction, the fusion protein has been suggested to interact with p300 through the NHR1 domain of the ETO moiety; RUNX1 self-acetylation is maintained [141] but local histone acetylation has yet to be proven. Chromatin immunoprecipitation sequencing (ChIP-seq) on p300 and a wide range of chromatin markers showed indeed conflicting results. The study demonstrated hypoacetylated histones at RUNX1-ETO binding sites with p300 colocalization [142] and appears to be in competition with HDACs recruited by ETO NHR domains. Similarly to the p300 acetyltransferase, RUNX1 and RUNX1-ETO are able to modulate chromatin structure by recruiting type I arginine methyltransferases, such as protein arginine methyltransferase 1 (PRMT1), which monomethylate or asymmetrically dimethylate arginine residues on histones [143]. Methylation on histone H4 arginine 3 (H4R3) by PRMT1 generally correlates with transcription activation and promotes subsequent p300- and PRMT4-mediated (CARM1) acetylation and methylation of histone tails [144]. PRMT1 is shown to interact with RUNX1 in HeLa cells by co-immunoprecipitation assay [145] and with RUNX1-ETO by mass spectrometry subsequent to FLAG-tagged fusion protein immunoprecipitation as well as in Kasumi-1 cell line [146,147]. PRMT1 is the most predominant arginine methyltransferase in mammalian cells which functions as a coactivator for transcription activators [148] and regulates numerous cellular processes such as DNA damage response and cell cycle checkpoint [149]. In the same way as PRMT1, CARM1is shown using a mouse model to play an essential role in t(8;21) AML leukemogenesis but appears of less importance in normal hematopoietic stem and progenitor cell differentiation and proliferation. Strikingly in other cancer types, CARM1 specifically dimethylates H3R17 and H3R26 histone arginine residues and uses a broad range of substrates such as transcription factors (including RUNX1) and chromatin modifiers [144,150,151,152,153].

## 6. Higher-Order Chromatin Structure Differentially Regulates the RUNX1/RUNX1-ETO Balance

Although it has been suggested that binding of native RUNX1 with p300 and RUNX1-ETO with HDACs is a mutually exclusive event [154], the precise mechanisms by which the oncofusion protein deregulates the RUNX1 program and chromatin conformation remain unclear. Several studies explored the chromatin and expression state in the presence of RUNX1-ETO, for which purpose the Kasumi-1 cell line was widely used. Using chromatin immunoprecipitation assays, 60% of RUNX1-ETO peaks is shown to overlap with RUNX1. The same study demonstrated that after RUNX1-ETO knockdown, the number of RUNX1 peaks increased and formed large number of de novo RUNX1 binding sites [155].

More recent work exhibited that 39% of RUNX1-ETO peaks overlap those of the N-CoR repressor, whereas only 22% overlap those of the p300 activator [156]. Although these data support the long-standing hypothesis that RUNX1-ETO acts as a dominant-negative regulator of RUNX1 targets, these also suggest that the fusion protein acts on a higher order of chromatin structure. Indeed, RUNX1 and RUNX1-ETO largely occupy promoter and distal sites [156] but display an unaltered balance of p300/HDAC at these regions [134]. Also, a study in mouse embryonic stem cell line demonstrated that RUNX1 initiated chromatin unfolding in the PU.1 locus before the activation of histone marks [157] which presents chromatin structure regulation as the earliest regulatory event.

It is interesting to note that the PU.1 locus is governed by an upstream regulatory element (URE) which is responsible for enhancer-mediated regulation. Reporter assays with ectopic expression of RUNX1-ETO and t(8;21) AML patient samples showed that RUNX1-ETO is capable of decreasing PU.1 expression levels [158]. Additionally, in Kasumi-1 cells, ChIP-seq demonstrated that both RUNX1 and RUNX1-ETO bind the PU.1 URE [155]. As demonstrated in mice, removal of the URE significantly decreases PU.1 levels but also induces leukemia [159]. Finally, our work evidenced by chromosomal conformation capture that loss of native RUNX1 binding to the URE of PU.1 in mice decreased the interaction between the URE and PU.1 promoter [90]. Given the evidence, it is tempting to hypothesize that RUNX1-ETO disrupts essential regulatory loop formation as evidenced here in the PU.1 locus. During the writing of this review, cyclin D2 (CCND2), a cell cycle regulator with similar chromosomal looping properties has been identified as an important player in leukemic propagation in t(8;21) AML. Knockdown of RUNX1-ETO decreased its binding to its -30 kb regulatory element, but more interestingly, revealed the binding of native RUNX1 instead. DNaseI accessibility, H3K9ac marker for active transcription, RNA-sequencing, and genome-wide chromosome conformation capture confirmed this finding [160]. Although RUNX1-ETO is often associated to repressed gene expression, here the fusion protein is shown to activate CCND2 by interfering with an intergenic negative regulatory element.

CCND2 and PU.1 have respectively increased and decreased expression in the presence of RUNX1-ETO. Both loci are relevant gene models to investigate chromatin disruption and altered gene expression by regulatory elements interference by RUNX1-ETO.

## 7. Summary and Final Remarks

Because of the almost complete ETO moiety, the oncofusion protein inherited the ability to interact with and recruit a wide range of repressors. RUNX1-ETO is thus commonly understood to suppress native RUNX1 activity. Although true, early studies after the discovery of the fusion protein failed to identify its activation function which is supported by a growing body of literature. Past efforts in providing a comprehensive picture of the genome-wide localization and the target genes of RUNX1-ETO have been inconclusive in understanding the underlying mechanism by which it deregulates native RUNX1. Both share similarities and, in recent years, research focused on the epigenetic factors to explain their differences. The presence of the t(8;21) translocation was associated with impaired DNA repair mechanism which compromises genomic integrity, thus favoring a mutator phenotype. Among an ever-increasing pool of mutated factors, regulators of DNA methylation were also frequently found in t(8;21) AML. Together with the alteration of both histone markers and distal enhancer regulation, RUNX1-ETO is depicted here as disrupting the chromatin structure. Epigenetic studies on the fusion protein uncovered new mechanisms into contributing to leukemogenesis and hopefully will translate into clinical applications.

## References

[1] Hanahan D., Weinberg R.A. (2011). Hallmarks of cancer: The next generation. Cell.

[2] Rowley J.D. (1973). Identificaton of a translocation with quinacrine fluorescence in a patient with acute leukemia. Ann. Genet..

[3] Grimwade D., Hills R.K., Moorman A.V., Walker H., Chatters S., Goldstone A.H., Wheatley K., Harrison C.J., Burnett A.K. (2010). Refinement of cytogenetic classification in acute myeloid leukemia: Determination of prognostic significance of rare recurring chromosomal abnormalities among 5876 younger adult patients treated in the united kingdom medical research council trials. Blood.

[4] Lin S., Mulloy J.C., Goyama S. (2017). RUNX1-ETO leukemia. Adv. Exp. Med. Biol..

[5] Sood R., Kamikubo Y., Liu P. (2017). Role of RUNX1 in hematological malignancies. Blood.

[6] Rowley J.D. (1984). Biological implications of consistent chromosome rearrangements in leukemia and lymphoma. Cancer Res..

[7] Gergen J.P., Wieschaus E.F. (1985). The localized requirements for a gene affecting segmentation in drosophila: Analysis of larvae mosaic for runt. Dev. Biol..

[8] Van Wijnen A.J., Stein G.S., Gergen J.P., Groner Y., Hiebert S.W., Ito Y., Liu P., Neil J.C., Ohki M., Speck N. (2004). Nomenclature for runt-related (RUNX) proteins. Oncogene.

[9] Higuchi M., O’Brien D., Kumaravelu P., Lenny N., Yeoh E.J., Downing J.R. (2002). Expression of a conditional AML1-ETO oncogene bypasses embryonic lethality and establishes a murine model of human t(8;21) acute myeloid leukemia. Cancer Cell.

[10] Yuan Y., Zhou L., Miyamoto T., Iwasaki H., Harakawa N., Hetherington C.J., Burel S.A., Lagasse E., Weissman I.L., Akashi K. (2001). AML1-ETO expression is directly involved in the development of acute myeloid leukemia in the presence of additional mutations. Proc. Natl. Acad. Sci. USA.

[11] Miyoshi H., Shimizu K., Kozu T., Maseki N., Kaneko Y., Ohki M. (1991). T(8;21) breakpoints on chromosome 21 in acute myeloid leukemia are clustered within a limited region of a single gene, AML1. Proc. Natl. Acad. Sci. USA.

[12] Lacaud G., Kouskoff V., Trumble A., Schwantz S., Keller G. (2004). Haploinsufficiency of RUNX1 results in the acceleration of mesodermal development and hemangioblast specification upon in vitro differentiation of es cells. Blood.

[13] North T., Gu T.L., Stacy T., Wang Q., Howard L., Binder M., Marin-Padilla M., Speck N.A. (1999). Cbfa2 is required for the formation of intra-aortic hematopoietic clusters. Development.

[14] Palis J., Robertson S., Kennedy M., Wall C., Keller G. (1999). Development of erythroid and myeloid progenitors in the yolk sac and embryo proper of the mouse. Development.

[15] Ferkowicz M.J., Yoder M.C. (2005). Blood island formation: Longstanding observations and modern interpretations. Exp. Hematol..

[16] Kieusseian A., Brunet de la Grange P., Burlen-Defranoux O., Godin I., Cumano A. (2012). Immature hematopoietic stem cells undergo maturation in the fetal liver. Development.

[17] Rybtsov S., Ivanovs A., Zhao S., Medvinsky A. (2016). Concealed expansion of immature precursors underpins acute burst of adult hsc activity in foetal liver. Development.

[18] North T.E., Stacy T., Matheny C.J., Speck N.A., de Bruijn M.F. (2004). RUNX1 is expressed in adult mouse hematopoietic stem cells and differentiating myeloid and lymphoid cells, but not in maturing erythroid cells. Stem Cells.

[19] Lorsbach R.B., Moore J., Ang S.O., Sun W., Lenny N., Downing J.R. (2004). Role of RUNX1 in adult hematopoiesis: Analysis of RUNX1-IRES-GFP knock-in mice reveals differential lineage expression. Blood.

[20] Okuda T., van Deursen J., Hiebert S.W., Grosveld G., Downing J.R. (1996). AML1, the target of multiple chromosomal translocations in human leukemia, is essential for normal fetal liver hematopoiesis. Cell.

[21] Wang Q., Stacy T., Miller J.D., Lewis A.F., Gu T.L., Huang X., Bushweller J.H., Bories J.C., Alt F.W., Ryan G. (1996). The CBFbeta subunit is essential for CBFalpha2 (AML1) function in vivo. Cell.

[22] Takakura N., Watanabe T., Suenobu S., Yamada Y., Noda T., Ito Y., Satake M., Suda T. (2000). A role for hematopoietic stem cells in promoting angiogenesis. Cell.

[23] Wang Q., Stacy T., Binder M., Marin-Padilla M., Sharpe A.H., Speck N.A. (1996). Disruption of the cbfa2 gene causes necrosis and hemorrhaging in the central nervous system and blocks definitive hematopoiesis. Proc. Natl. Acad. Sci. USA.

[24] Muller A.M., Medvinsky A., Strouboulis J., Grosveld F., Dzierzak E. (1994). Development of hematopoietic stem cell activity in the mouse embryo. Immunity.

[25] Cai Z., de Bruijn M., Ma X., Dortland B., Luteijn T., Downing R.J., Dzierzak E. (2000). Haploinsufficiency of AML1 affects the temporal and spatial generation of hematopoietic stem cells in the mouse embryo. Immunity.

[26] Goetz T.L., Gu T.L., Speck N.A., Graves B.J. (2000). Auto-inhibition of Ets-1 is counteracted by DNA binding cooperativity with core-binding factor alpha2. Mol. Cell. Biol..

[27] Imperato M.R., Cauchy P., Obier N., Bonifer C. (2015). The RUNX1-PU.1 axis in the control of hematopoiesis. Int. J. Hematol..

[28] Elagib K.E., Racke F.K., Mogass M., Khetawat R., Delehanty L.L., Goldfarb A.N. (2003). RUNX1 and GATA-1 coexpression and cooperation in megakaryocytic differentiation. Blood.

[29] Takahashi A., Satake M., Yamaguchi-Iwai Y., Bae S.C., Lu J., Maruyama M., Zhang Y.W., Oka H., Arai N., Arai K. (1995). Positive and negative regulation of granulocyte-macrophage colony-stimulating factor promoter activity by AML1-related transcription factor, PEBP2. Blood.

[30] Zhang D.E., Fujioka K., Hetherington C.J., Shapiro L.H., Chen H.M., Look A.T., Tenen D.G. (1994). Identification of a region which directs the monocytic activity of the colony-stimulating factor 1 (macrophage colony-stimulating factor) receptor promoter and binds PEBP2/CBF (AML1). Mol. Cell. Biol..

[31] Zhang Y., Derynck R. (2000). Transcriptional regulation of the transforming growth factor-beta -inducible mouse germ line Ig alpha constant region gene by functional cooperation of Smad, CREB, and AML family members. J. Biol. Chem..

[32] Libermann T.A., Pan Z., Akbarali Y., Hetherington C.J., Boltax J., Yergeau D.A., Zhang D.E. (1999). AML1 (CBFα2) cooperates with b cell-specific activating protein (BSAP/PAX5) in activation of the B cell-specific BLK gene promoter. J. Biol. Chem..

[33] Sun W., Graves B.J., Speck N.A. (1995). Transactivation of the moloney murine leukemia virus and T-cell receptor beta-chain enhancers by cbf and ets requires intact binding sites for both proteins. J. Virol..

[34] Puig-Kroger A., Sanchez-Elsner T., Ruiz N., Andreu E.J., Prosper F., Jensen U.B., Gil J., Erickson P., Drabkin H., Groner Y. (2003). RUNX/AML and C/EBP factors regulate cd11a integrin expression in myeloid cells through overlapping regulatory elements. Blood.

[35] De Braekeleer E., Douet-Guilbert N., Morel F., Le Bris M.J., Ferec C., De Braekeleer M. (2011). RUNX1 translocations and fusion genes in malignant hemopathies. Future Oncol. (London, England).

[36] Gaidzik V.I., Bullinger L., Schlenk R.F., Zimmermann A.S., Rock J., Paschka P., Corbacioglu A., Krauter J., Schlegelberger B., Ganser A. (2011). RUNX1 mutations in acute myeloid leukemia: Results from a comprehensive genetic and clinical analysis from the aml study group. J. Clin. Oncol..

[37] Schnittger S., Dicker F., Kern W., Wendland N., Sundermann J., Alpermann T., Haferlach C., Haferlach T. (2011). RUNX1 mutations are frequent in de novo aml with noncomplex karyotype and confer an unfavorable prognosis. Blood.

[38] Tsuzuki S., Hong D., Gupta R., Matsuo K., Seto M., Enver T. (2007). Isoform-specific potentiation of stem and progenitor cell engraftment by AML1/RUNX1. PLoS Med..

[39] Ran D., Shia W.J., Lo M.C., Fan J.B., Knorr D.A., Ferrell P.I., Ye Z., Yan M., Cheng L., Kaufman D.S. (2013). RUNX1a enhances hematopoietic lineage commitment from human embryonic stem cells and inducible pluripotent stem cells. Blood.

[40] Challen G.A., Goodell M.A. (2010). RUNX1 isoforms show differential expression patterns during hematopoietic development but have similar functional effects in adult hematopoietic stem cells. Exp. Hematol..

[41] Draper J.E., Sroczynska P., Tsoulaki O., Leong H.S., Fadlullah M.Z., Miller C., Kouskoff V., Lacaud G. (2016). RUNX1b expression is highly heterogeneous and distinguishes megakaryocytic and erythroid lineage fate in adult mouse hematopoiesis. PLoS Genet..

[42] Draper J.E., Sroczynska P., Leong H.S., Fadlullah M.Z.H., Miller C., Kouskoff V., Lacaud G. (2017). Mouse RUNX1c regulates premegakaryocytic/erythroid output and maintains survival of megakaryocyte progenitors. Blood.

[43] Imai Y., Kurokawa M., Tanaka K., Friedman A.D., Ogawa S., Mitani K., Yazaki Y., Hirai H. (1998). Tle, the human homolog of groucho, interacts with AML1 and acts as a repressor of AML1-induced transactivation. Biochem. Biophys. Res. Commun..

[44] Meyers S., Downing J.R., Hiebert S.W. (1993). Identification of AML-1 and the (8;21) translocation protein (AML-1/ETO) as sequence-specific DNA-binding proteins: The runt homology domain is required for DNA binding and protein-protein interactions. Mol. Cell. Biol..

[45] Golling G., Li L., Pepling M., Stebbins M., Gergen J.P. (1996). Drosophila homologs of the proto-oncogene product PEBP2/CBF beta regulate the DNA-binding properties of runt. Mol. Cell. Biol..

[46] Blake T., Adya N., Kim C.H., Oates A.C., Zon L., Chitnis A., Weinstein B.M., Liu P.P. (2000). Zebrafish homolog of the leukemia gene CBFB: Its expression during embryogenesis and its relationship to scl and gata-1 in hematopoiesis. Blood.

[47] Ogawa E., Maruyama M., Kagoshima H., Inuzuka M., Lu J., Satake M., Shigesada K., Ito Y. (1993). PEBP2/PEA2 represents a family of transcription factors homologous to the products of the drosophila runt gene and the human AML1 gene. Proc. Natl. Acad. Sci. USA.

[48] Wang S., Wang Q., Crute B.E., Melnikova I.N., Keller S.R., Speck N.A. (1993). Cloning and characterization of subunits of the T-cell receptor and murine leukemia virus enhancer core-binding factor. Mol. Cell. Biol..

[49] Tahirov T.H., Bushweller J. (2017). Structure and biophysics of CBFβ/RUNX and its translocation products. Adv. Exp. Med. Biol..

[50] Huang G., Shigesada K., Ito K., Wee H.J., Yokomizo T., Ito Y. (2001). Dimerization with PEBP2β protects RUNX1/AML1 from ubiquitin-proteasome-mediated degradation. EMBO J..

[51] Wee H.J., Voon D.C., Bae S.C., Ito Y. (2008). PEBP2-β/CBF-β-dependent phosphorylation of RUNX1 and p300 by HIPK2: Implications for leukemogenesis. Blood.

[52] Zhang D.E., Hetherington C.J., Meyers S., Rhoades K.L., Larson C.J., Chen H.M., Hiebert S.W., Tenen D.G. (1996). CCAAT enhancer-binding protein (C/EBP) and AML1 (CBF alpha2) synergistically activate the macrophage colony-stimulating factor receptor promoter. Mol. Cell. Biol..

[53] Petrovick M.S., Hiebert S.W., Friedman A.D., Hetherington C.J., Tenen D.G., Zhang D.E. (1998). Multiple functional domains of AML1: PU.1 and C/EBPalpha synergize with different regions of AML1. Mol. Cell. Biol..

[54] Imai Y., Kurokawa M., Yamaguchi Y., Izutsu K., Nitta E., Mitani K., Satake M., Noda T., Ito Y., Hirai H. (2004). The corepressor mSin3A regulates phosphorylation-induced activation, intranuclear location, and stability of AML1. Mol. Cell. Biol..

[55] Kitabayashi I., Yokoyama A., Shimizu K., Ohki M. (1998). Interaction and functional cooperation of the leukemia-associated factors AML1 and p300 in myeloid cell differentiation. EMBO J..

[56] Huang H., Yu M., Akie T.E., Moran T.B., Woo A.J., Tu N., Waldon Z., Lin Y.Y., Steen H., Cantor A.B. (2009). Differentiation-dependent interactions between RUNX-1 and fli-1 during megakaryocyte development. Mol. Cell. Biol..

[57] Michaud J., Scott H.S., Escher R. (2003). AML1 interconnected pathways of leukemogenesis. Cancer Investig..

[58] Ito Y. (2004). Oncogenic potential of the RUNX gene family: ‘Overview’. Oncogene.

[59] Miyoshi H., Kozu T., Shimizu K., Enomoto K., Maseki N., Kaneko Y., Kamada N., Ohki M. (1993). The t(8;21) translocation in acute myeloid leukemia results in production of an AML1-MTG8 fusion transcript. EMBO J..

[60] Muller A.M., Duque J., Shizuru J.A., Lubbert M. (2008). Complementing mutations in core binding factor leukemias: From mouse models to clinical applications. Oncogene.

[61] Grimwade D., Walker H., Oliver F., Wheatley K., Harrison C., Harrison G., Rees J., Hann I., Stevens R., Burnett A. (1998). The importance of diagnostic cytogenetics on outcome in aml: Analysis of 1,612 patients entered into the MRC AML 10 trial. The medical research council adult and children’s leukaemia working parties. Blood.

[62] Byrd J.C., Mrozek K., Dodge R.K., Carroll A.J., Edwards C.G., Arthur D.C., Pettenati M.J., Patil S.R., Rao K.W., Watson M.S. (2002). Pretreatment cytogenetic abnormalities are predictive of induction success, cumulative incidence of relapse, and overall survival in adult patients with de novo acute myeloid leukemia: Results from cancer and leukemia group b (CALGB 8461). Blood.

[63] Kuchenbauer F., Schnittger S., Look T., Gilliland G., Tenen D., Haferlach T., Hiddemann W., Buske C., Schoch C. (2006). Identification of additional cytogenetic and molecular genetic abnormalities in acute myeloid leukaemia with t(8;21)/AML1-ETO. Br. J. Haematol..

[64] Prebet T., Boissel N., Reutenauer S., Thomas X., Delaunay J., Cahn J.Y., Pigneux A., Quesnel B., Witz F., Thepot S. (2009). Acute myeloid leukemia with translocation (8;21) or inversion (16) in elderly patients treated with conventional chemotherapy: A collaborative study of the french cbf-aml intergroup. J. Clin. Oncol..

[65] Wolford J.K., Prochazka M. (1998). Structure and expression of the human MTG8/ETO gene. Gene.

[66] Kozu T., Fukuyama T., Yamami T., Akagi K., Kaneko Y. (2005). Mynd-less splice variants of AML1-MTG8 (RUNX1-CBFA2T1) are expressed in leukemia with t(8;21). Gene. Chromosome Cancer.

[67] Davis J.N., McGhee L., Meyers S. (2003). The ETO (MTG8) gene family. Gene.

[68] Feinstein P.G., Kornfeld K., Hogness D.S., Mann R.S. (1995). Identification of homeotic target genes in drosophila melanogaster including nervy, a proto-oncogene homologue. Genetics.

[69] Zhang J., Hug B.A., Huang E.Y., Chen C.W., Gelmetti V., Maccarana M., Minucci S., Pelicci P.G., Lazar M.A. (2001). Oligomerization of eto is obligatory for corepressor interaction. Mol. Cell. Biol..

[70] Lutterbach B., Westendorf J.J., Linggi B., Patten A., Moniwa M., Davie J.R., Huynh K.D., Bardwell V.J., Lavinsky R.M., Rosenfeld M.G. (1998). ETO, a target of t(8;21) in acute leukemia, interacts with the n-cor and msin3 corepressors. Mol. Cell. Biol..

[71] Wang J., Hoshino T., Redner R.L., Kajigaya S., Liu J.M. (1998). ETO, fusion partner in t(8;21) acute myeloid leukemia, represses transcription by interaction with the human N-CoR/mSin3/HDAC1 complex. Proc. Natl. Acad. Sci. USA.

[72] Erickson P.F., Robinson M., Owens G., Drabkin H.A. (1994). The ETO portion of acute myeloid leukemia t(8;21) fusion transcript encodes a highly evolutionarily conserved, putative transcription factor. Cancer Res..

[73] Zhang J., Kalkum M., Yamamura S., Chait B.T., Roeder R.G. (2004). E protein silencing by the leukemogenic AML1-ETO fusion protein. Science.

[74] Plevin M.J., Zhang J., Guo C., Roeder R.G., Ikura M. (2006). The acute myeloid leukemia fusion protein AML1-ETO targets e proteins via a paired amphipathic helix-like TBP-associated factor homology domain. Proc. Natl. Acad. Sci. USA.

[75] Kitabayashi I., Ida K., Morohoshi F., Yokoyama A., Mitsuhashi N., Shimizu K., Nomura N., Hayashi Y., Ohki M. (1998). The AML1-MTG8 leukemic fusion protein forms a complex with a novel member of the MTG8(ETO/CDR) family, mtgr1. Mol. Cell. Biol..

[76] Liu Y., Cheney M.D., Gaudet J.J., Chruszcz M., Lukasik S.M., Sugiyama D., Lary J., Cole J., Dauter Z., Minor W. (2006). The tetramer structure of the nervy homology two domain, NHR2, is critical for AML1/ETO’S activity. Cancer Cell.

[77] Hug B.A., Lazar M.A. (2004). ETO interacting proteins. Oncogene.

[78] Odaka Y., Mally A., Elliott L.T., Meyers S. (2000). Nuclear import and subnuclear localization of the proto-oncoprotein ETO (MTG8). Oncogene.

[79] Barseguian K., Lutterbach B., Hiebert S.W., Nickerson J., Lian J.B., Stein J.L., van Wijnen A.J., Stein G.S. (2002). Multiple subnuclear targeting signals of the leukemia-related AML1/ETO and ETO repressor proteins. Proc. Natl. Acad. Sci. USA.

[80] Tighe J.E., Calabi F. (1995). T(8;21) breakpoints are clustered between alternatively spliced exons of MTG8. Clin. Sci. (Lond.).

[81] Zhang Y., Strissel P., Strick R., Chen J., Nucifora G., Le Beau M.M., Larson R.A., Rowley J.D. (2002). Genomic DNA breakpoints in AML1/RUNX1 and ETO cluster with topoisomerase ii DNA cleavage and dnase i hypersensitive sites in t(8;21) leukemia. Proc. Natl. Acad. Sci. USA.

[82] Ugarte G.D., Vargas M.F., Medina M.A., Leon P., Necunir D., Elorza A.A., Gutierrez S.E., Moon R.T., Loyola A., De Ferrari G.V. (2015). Wnt signaling induces transcription, spatial proximity, and translocation of fusion gene partners in human hematopoietic cells. Blood.

[83] Kwok C., Zeisig B.B., Qiu J., Dong S., So C.W. (2009). Transforming activity of AML1-ETO is independent of CBFbeta and eto interaction but requires formation of homo-oligomeric complexes. Proc. Natl. Acad. Sci. USA.

[84] Hug B.A., Lee S.Y., Kinsler E.L., Zhang J., Lazar M.A. (2002). Cooperative function of AML1-ETO corepressor recruitment domains in the expansion of primary bone marrow cells. Cancer Res..

[85] DeKelver R.C., Yan M., Ahn E.Y., Shia W.J., Speck N.A., Zhang D.E. (2013). Attenuation of AML1-ETO cellular dysregulation correlates with increased leukemogenic potential. Blood.

[86] Yan M., Kanbe E., Peterson L.F., Boyapati A., Miao Y., Wang Y., Chen I.M., Chen Z., Rowley J.D., Willman C.L. (2006). A previously unidentified alternatively spliced isoform of t(8;21) transcript promotes leukemogenesis. Nat. Med..

[87] Tang J.L., Hou H.A., Chen C.Y., Liu C.Y., Chou W.C., Tseng M.H., Huang C.F., Lee F.Y., Liu M.C., Yao M. (2009). AML1/RUNX1 mutations in 470 adult patients with de novo acute myeloid leukemia: Prognostic implication and interaction with other gene alterations. Blood.

[88] Ben-Ami O., Friedman D., Leshkowitz D., Goldenberg D., Orlovsky K., Pencovich N., Lotem J., Tanay A., Groner Y. (2013). Addiction of t(8;21) and inv(16) acute myeloid leukemia to native RUNX1. Cell Rep..

[89] Goyama S., Schibler J., Cunningham L., Zhang Y., Rao Y., Nishimoto N., Nakagawa M., Olsson A., Wunderlich M., Link K.A. (2013). Transcription factor RUNX1 promotes survival of acute myeloid leukemia cells. J. Clin. Investig..

[90] Staber P.B., Zhang P., Ye M., Welner R.S., Levantini E., Di Ruscio A., Ebralidze A.K., Bach C., Zhang H., Zhang J. (2014). The RUNX-PU.1 pathway preserves normal and AML/ETO9a leukemic stem cells. Blood.

[91] Li Y., Wang H., Wang X., Jin W., Tan Y., Fang H., Chen S., Chen Z., Wang K. (2016). Genome-wide studies identify a novel interplay between AML1 and AML1/ETO in t(8;21) acute myeloid leukemia. Blood.

[92] Lindahl T., Barnes D.E. (2000). Repair of endogenous DNA damage. Cold Spring Harbor Symposia on Quantitative Biology.

[93] Kastan M.B. (2008). DNA damage responses: Mechanisms and roles in human disease: 2007 g.H.A. Clowes memorial award lecture. Mol. Cancer Res. MCR.

[94] Jackson S.P., Bartek J. (2009). The DNA-damage response in human biology and disease. Nature.

[95] Begg A.C., Stewart F.A., Vens C. (2011). Strategies to improve radiotherapy with targeted drugs. Nat. Rev. Cancer.

[96] Alcalay M., Meani N., Gelmetti V., Fantozzi A., Fagioli M., Orleth A., Riganelli D., Sebastiani C., Cappelli E., Casciari C. (2003). Acute myeloid leukemia fusion proteins deregulate genes involved in stem cell maintenance and DNA repair. J. Clin. Investig..

[97] Krejci O., Wunderlich M., Geiger H., Chou F.S., Schleimer D., Jansen M., Andreassen P.R., Mulloy J.C. (2008). P53 signaling in response to increased DNA damage sensitizes AML1-ETO cells to stress-induced death. Blood.

[98] Forster V.J., Nahari M.H., Martinez-Soria N., Bradburn A.K., Ptasinska A., Assi S.A., Fordham S.E., McNeil H., Bonifer C., Heidenreich O. (2016). The leukemia-associated RUNX1/ETO oncoprotein confers a mutator phenotype. Leukemia.

[99] Liddiard K., Hills R., Burnett A.K., Darley R.L., Tonks A. (2010). OGG1 is a novel prognostic indicator in acute myeloid leukaemia. Oncogene.

[100] Esposito M.T., Zhao L., Fung T.K., Rane J.K., Wilson A., Martin N., Gil J., Leung A.Y., Ashworth A., So C.W. (2015). Synthetic lethal targeting of oncogenic transcription factors in acute leukemia by parp inhibitors. Nat. Med..

[101] Satoh Y., Matsumura I., Tanaka H., Harada H., Harada Y., Matsui K., Shibata M., Mizuki M., Kanakura Y. (2012). C-terminal mutation of RUNX1 attenuates the DNA-damage repair response in hematopoietic stem cells. Leukemia.

[102] Zuber J., Radtke I., Pardee T.S., Zhao Z., Rappaport A.R., Luo W., McCurrach M.E., Yang M.M., Dolan M.E., Kogan S.C. (2009). Mouse models of human aml accurately predict chemotherapy response. Genes Dev..

[103] Bullinger L., Rucker F.G., Kurz S., Du J., Scholl C., Sander S., Corbacioglu A., Lottaz C., Krauter J., Frohling S. (2007). Gene-expression profiling identifies distinct subclasses of core binding factor acute myeloid leukemia. Blood.

[104] Mrozek K., Bloomfield C.D. (2008). Clinical significance of the most common chromosome translocations in adult acute myeloid leukemia. J. Natl. Cancer Inst. Monogr..

[105] Krauth M.T., Eder C., Alpermann T., Bacher U., Nadarajah N., Kern W., Haferlach C., Haferlach T., Schnittger S. (2014). High number of additional genetic lesions in acute myeloid leukemia with t(8;21)/RUNX1-RUNX1t1: Frequency and impact on clinical outcome. Leukemia.

[106] Matsuura S., Yan M., Lo M.C., Ahn E.Y., Weng S., Dangoor D., Matin M., Higashi T., Feng G.S., Zhang D.E. (2012). Negative effects of gm-csf signaling in a murine model of t(8;21)-induced leukemia. Blood.

[107] Dayyani F., Wang J., Yeh J.R., Ahn E.Y., Tobey E., Zhang D.E., Bernstein I.D., Peterson R.T., Sweetser D.A. (2008). Loss of TLE1 and TLE4 from the del(9q) commonly deleted region in aml cooperates with AML1-ETO to affect myeloid cell proliferation and survival. Blood.

[108] Marcucci G., Mrozek K., Ruppert A.S., Maharry K., Kolitz J.E., Moore J.O., Mayer R.J., Pettenati M.J., Powell B.L., Edwards C.G. (2005). Prognostic factors and outcome of core binding factor acute myeloid leukemia patients with t(8;21) differ from those of patients with inv(16): A cancer and leukemia group b study. J. Clin. Oncol..

[109] Wakita S., Yamaguchi H., Miyake K., Mitamura Y., Kosaka F., Dan K., Inokuchi K. (2011). Importance of c-kit mutation detection method sensitivity in prognostic analyses of t(8;21)(q22;q22) acute myeloid leukemia. Leukemia.

[110] Fu L., Fu H., Tian L., Xu K., Hu K., Wang J., Wang J., Jing H., Shi J., Ke X. (2016). High expression of RUNX1 is associated with poorer outcomes in cytogenetically normal acute myeloid leukemia. Oncotarget.

[111] Bird A.P. (1986). CPG-rich islands and the function of DNA methylation. Nature.

[112] Ji H., Ehrlich L.I., Seita J., Murakami P., Doi A., Lindau P., Lee H., Aryee M.J., Irizarry R.A., Kim K. (2010). Comprehensive methylome map of lineage commitment from haematopoietic progenitors. Nature.

[113] Bock C., Beerman I., Lien W.H., Smith Z.D., Gu H., Boyle P., Gnirke A., Fuchs E., Rossi D.J., Meissner A. (2012). DNA methylation dynamics during in vivo differentiation of blood and skin stem cells. Mol. Cell.

[114] Messerschmidt D.M., Knowles B.B., Solter D. (2014). DNA methylation dynamics during epigenetic reprogramming in the germline and preimplantation embryos. Genes Dev..

[115] Suzuki T., Shimizu Y., Furuhata E., Maeda S., Kishima M., Nishimura H., Enomoto S., Hayashizaki Y., Suzuki H. (2017). RUNX1 regulates site specificity of DNA demethylation by recruitment of DNA demethylation machineries in hematopoietic cells. Blood Adv..

[116] Pastor W.A., Aravind L., Rao A. (2013). TETonic shift: Biological roles of TET proteins in DNA demethylation and transcription. Nat. Rev. Mol. Cell Biol..

[117] Solary E., Bernard O.A., Tefferi A., Fuks F., Vainchenker W. (2014). The ten-eleven translocation-2 (tet2) gene in hematopoiesis and hematopoietic diseases. Leukemia.

[118] Baylin S.B., Jones P.A. (2011). A decade of exploring the cancer epigenome—Biological and translational implications. Nat. Rev. Cancer.

[119] Weissmann S., Alpermann T., Grossmann V., Kowarsch A., Nadarajah N., Eder C., Dicker F., Fasan A., Haferlach C., Haferlach T. (2012). Landscape of TET2 mutations in acute myeloid leukemia. Leukemia.

[120] Faber Z.J., Chen X., Gedman A.L., Boggs K., Cheng J., Ma J., Radtke I., Chao J.R., Walsh M.P., Song G. (2016). The genomic landscape of core-binding factor acute myeloid leukemias. Nat. Genet..

[121] Rasmussen K.D., Jia G., Johansen J.V., Pedersen M.T., Rapin N., Bagger F.O., Porse B.T., Bernard O.A., Christensen J., Helin K. (2015). Loss of TET2 in hematopoietic cells leads to DNA hypermethylation of active enhancers and induction of leukemogenesis. Genes Dev..

[122] Reitman Z.J., Yan H. (2010). Isocitrate dehydrogenase 1 and 2 mutations in cancer: Alterations at a crossroads of cellular metabolism. J. Natl. Cancer Inst..

[123] Figueroa M.E., Lugthart S., Li Y., Erpelinck-Verschueren C., Deng X., Christos P.J., Schifano E., Booth J., van Putten W., Skrabanek L. (2010). DNA methylation signatures identify biologically distinct subtypes in acute myeloid leukemia. Cancer Cell.

[124] Gaidzik V.I., Paschka P., Spath D., Habdank M., Kohne C.H., Germing U., von Lilienfeld-Toal M., Held G., Horst H.A., Haase D. (2012). TET2 mutations in acute myeloid leukemia (AML): Results from a comprehensive genetic and clinical analysis of the aml study group. J. Clin. Oncol..

[125] Okano M., Xie S., Li E. (1998). Cloning and characterization of a family of novel mammalian DNA (cytosine-5) methyltransferases. Nat. Genet..

[126] Okano M., Bell D.W., Haber D.A., Li E. (1999). DNA methyltransferases Dnmt3a and Dnmt3b are essential for de novo methylation and mammalian development. Cell.

[127] Liu S., Shen T., Huynh L., Klisovic M.I., Rush L.J., Ford J.L., Yu J., Becknell B., Li Y., Liu C. (2005). Interplay of RUNX1/mtg8 and DNA methyltransferase 1 in acute myeloid leukemia. Cancer Res..

[128] Zhou L., Fu L., Lv N., Liu J., Li Y., Chen X., Xu Q., Chen G., Pang B., Wang L. (2018). Methylation-associated silencing of BASP1 contributes to leukemogenesis in t(8;21) acute myeloid leukemia. Exp. Mol. Med..

[129] Gao X.N., Yan F., Lin J., Gao L., Lu X.L., Wei S.C., Shen N., Pang J.X., Ning Q.Y., Komeno Y. (2015). AML1/ETO cooperates with HIF1alpha to promote leukemogenesis through DNMT3a transactivation. Leukemia.

[130] Spitz F., Furlong E.E. (2012). Transcription factors: From enhancer binding to developmental control. Nat. Rev. Genet..

[131] Meyers S., Lenny N., Hiebert S.W. (1995). The t(8;21) fusion protein interferes with AML-1B-dependent transcriptional activation. Mol. Cell. Biol..

[132] Gelmetti V., Zhang J., Fanelli M., Minucci S., Pelicci P.G., Lazar M.A. (1998). Aberrant recruitment of the nuclear receptor corepressor-histone deacetylase complex by the acute myeloid leukemia fusion partner eto. Mol. Cell. Biol..

[133] Amann J.M., Nip J., Strom D.K., Lutterbach B., Harada H., Lenny N., Downing J.R., Meyers S., Hiebert S.W. (2001). ETO, a target of t(8;21) in acute leukemia, makes distinct contacts with multiple histone deacetylases and binds msin3a through its oligomerization domain. Mol. Cell. Biol..

[134] Mandoli A., Singh A.A., Prange K.H.M., Tijchon E., Oerlemans M., Dirks R., Ter Huurne M., Wierenga A.T.J., Janssen-Megens E.M., Berentsen K. (2016). The hematopoietic transcription factors RUNX1 and erg prevent AML1-ETO oncogene overexpression and onset of the apoptosis program in t(8;21) AMLs. Cell Rep..

[135] Gottlicher M., Minucci S., Zhu P., Kramer O.H., Schimpf A., Giavara S., Sleeman J.P., Lo Coco F., Nervi C., Pelicci P.G. (2001). Valproic acid defines a novel class of hdac inhibitors inducing differentiation of transformed cells. EMBO J..

[136] Blobel G.A. (2000). Creb-binding protein and p300: Molecular integrators of hematopoietic transcription. Blood.

[137] Kasper L.H., Boussouar F., Ney P.A., Jackson C.W., Rehg J., van Deursen J.M., Brindle P.K. (2002). A transcription-factor-binding surface of coactivator p300 is required for haematopoiesis. Nature.

[138] Rebel V.I., Kung A.L., Tanner E.A., Yang H., Bronson R.T., Livingston D.M. (2002). Distinct roles for creb-binding protein and p300 in hematopoietic stem cell self-renewal. Proc. Natl. Acad. Sci. USA.

[139] Yamaguchi Y., Kurokawa M., Imai Y., Izutsu K., Asai T., Ichikawa M., Yamamoto G., Nitta E., Yamagata T., Sasaki K. (2004). AML1 is functionally regulated through p300-mediated acetylation on specific lysine residues. J. Biol. Chem..

[140] Yoshida H., Kitabayashi I. (2008). Chromatin regulation by AML1 complex. Int. J. Hematol..

[141] Wang L., Gural A., Sun X.J., Zhao X., Perna F., Huang G., Hatlen M.A., Vu L., Liu F., Xu H. (2011). The leukemogenicity of AML1-ETO is dependent on site-specific lysine acetylation. Science.

[142] Saeed S., Logie C., Francoijs K.J., Frige G., Romanenghi M., Nielsen F.G., Raats L., Shahhoseini M., Huynen M., Altucci L. (2012). Chromatin accessibility, p300, and histone acetylation define PML-RARalpha and AML1-ETO binding sites in acute myeloid leukemia. Blood.

[143] Tang J., Frankel A., Cook R.J., Kim S., Paik W.K., Williams K.R., Clarke S., Herschman H.R. (2000). Prmt1 is the predominant type i protein arginine methyltransferase in mammalian cells. J. Biol. Chem..

[144] An W., Kim J., Roeder R.G. (2004). Ordered cooperative functions of PRMT1, p300, and CARM1 in transcriptional activation by p53. Cell.

[145] Zhao X., Jankovic V., Gural A., Huang G., Pardanani A., Menendez S., Zhang J., Dunne R., Xiao A., Erdjument-Bromage H. (2008). Methylation of RUNX1 by PRMT1 abrogates sin3a binding and potentiates its transcriptional activity. Genes Dev..

[146] Shia W.J., Okumura A.J., Yan M., Sarkeshik A., Lo M.C., Matsuura S., Komeno Y., Zhao X., Nimer S.D., Yates J.R. (2012). PRMT1 interacts with AML1-ETO to promote its transcriptional activation and progenitor cell proliferative potential. Blood.

[147] Sun X.J., Wang Z., Wang L., Jiang Y., Kost N., Soong T.D., Chen W.Y., Tang Z., Nakadai T., Elemento O. (2013). A stable transcription factor complex nucleated by oligomeric AML1-ETO controls leukaemogenesis. Nature.

[148] Barrero M.J., Malik S. (2006). Two functional modes of a nuclear receptor-recruited arginine methyltransferase in transcriptional activation. Mol. Cell.

[149] Bedford M.T., Richard S. (2005). Arginine methylation an emerging regulator of protein function. Mol. Cell.

[150] Wang L., Zeng H., Wang Q., Zhao Z., Boyer T.G., Bian X., Xu W. (2015). MED12 methylation by carm1 sensitizes human breast cancer cells to chemotherapy drugs. Sci. Adv..

[151] Schurter B.T., Koh S.S., Chen D., Bunick G.J., Harp J.M., Hanson B.L., Henschen-Edman A., Mackay D.R., Stallcup M.R., Aswad D.W. (2001). Methylation of histone h3 by coactivator-associated arginine methyltransferase 1. Biochemistry.

[152] Shishkova E., Zeng H., Liu F., Kwiecien N.W., Hebert A.S., Coon J.J., Xu W. (2017). Global mapping of CARM1 substrates defines enzyme specificity and substrate recognition. Nat. Commun..

[153] Daujat S., Bauer U.M., Shah V., Turner B., Berger S., Kouzarides T. (2002). Crosstalk between CARM1 methylation and CBP acetylation on histone h3. Curr. Biol. CB.

[154] Ptasinska A., Assi S.A., Martinez-Soria N., Imperato M.R., Piper J., Cauchy P., Pickin A., James S.R., Hoogenkamp M., Williamson D. (2014). Identification of a dynamic core transcriptional network in t(8;21) aml that regulates differentiation block and self-renewal. Cell Rep..

[155] Ptasinska A., Assi S.A., Mannari D., James S.R., Williamson D., Dunne J., Hoogenkamp M., Wu M., Care M., McNeill H. (2012). Depletion of RUNX1/ETO in t(8;21) aml cells leads to genome-wide changes in chromatin structure and transcription factor binding. Leukemia.

[156] Trombly D.J., Whitfield T.W., Padmanabhan S., Gordon J.A., Lian J.B., van Wijnen A.J., Zaidi S.K., Stein J.L., Stein G.S. (2015). Genome-wide co-occupancy of AML1-ETO and N-CoR defines the t(8;21) AML signature in leukemic cells. BMC Genom..

[157] Hoogenkamp M., Lichtinger M., Krysinska H., Lancrin C., Clarke D., Williamson A., Mazzarella L., Ingram R., Jorgensen H., Fisher A. (2009). Early chromatin unfolding by RUNX1: A molecular explanation for differential requirements during specification versus maintenance of the hematopoietic gene expression program. Blood.

[158] Vangala R.K., Heiss-Neumann M.S., Rangatia J.S., Singh S.M., Schoch C., Tenen D.G., Hiddemann W., Behre G. (2003). The myeloid master regulator transcription factor PU.1 is inactivated by AML1-ETO in t(8;21) myeloid leukemia. Blood.

[159] Rosenbauer F., Wagner K., Kutok J.L., Iwasaki H., Le Beau M.M., Okuno Y., Akashi K., Fiering S., Tenen D.G. (2004). Acute myeloid leukemia induced by graded reduction of a lineage-specific transcription factor, PU.1. Nat. Genet..

[160] Martinez-Soria N., McKenzie L., Draper J., Ptasinska A., Issa H., Potluri S., Blair H.J., Pickin A., Isa A., Chin P.S. (2018). The oncogenic transcription factor RUNX1/ETO corrupts cell cycle regulation to drive leukemic transformation. Cancer Cell.

